# Proof-of-Concept of Microwave-Based Bladder State Detection Using Realistic Pelvic Models

**DOI:** 10.1109/OJEMB.2023.3305838

**Published:** 2023-08-16

**Authors:** Ali Farshkaran, Andrew Fry, Alex Raterink, Adam Santorelli, Emily Porter

**Affiliations:** Department of Electrical and Computer EngineeringThe University of Texas at Austin 12330 Austin TX 78712 USA; Department of Electrical and Computer EngineeringThe University of Texas at Austin 12330 Austin TX 78712 USA; Rice University 3990 Houston TX 77005 USA; Department of Biomedical EngineeringThe University of Texas at Austin 12330 Austin TX 78712 USA; Department of Electrical and Computer EngineeringThe University of Texas at Austin 12330 Austin TX 78712 USA; Department of Biomedical EngineeringMcGill University 5620 QC H3A 2B4 Canada

**Keywords:** Bladder state, microwave detection, pelvic region model, urinary incontinence

## Abstract

*Goal:* Urinary incontinence (UI) affects a significant proportion of the population and is associated with negative physical and psychological side-effects. Microwave-based technologies may have the potential to monitor bladder volume, providing a proactive, low-cost and non-invasive tool to support individuals with UI. *Methods:* Studies to date on microwave bladder monitoring have been limited to highly simplified computational and experimental scenarios. In this work, we study the most realistic models to date (both male and female), which incorporate dielectrically and anatomically representative tissues of the pelvic region. *Results:* We examine the ability of detecting bladder fullness through both reflection and transmission-based parameters and, for the first time, study the effect of urine permittivity. As a proof-of-concept of bladder state detection, we further investigate reconstructing differential radar images of the bladder with two different volumes of urine. *Conclusions:* The results indicate that there is strong potential for monitoring and detecting the bladder state using microwave measurements.

## I. Introduction

Urinary incontinence (UI) affects more than 200 million individuals worldwide, and is particularly prevalent in women, the elderly, those with spinal cord injuries, and children and young adults with intellectual disabilities [1], [2], [3], [4]. UI can have a significant negative impact on an individual's quality of life, independence and dignity [1], [2]. While UI can be caused by numerous conditions [5], in this work we focus on a subset that involve the lack of sensation that the bladder is full or the lack of ability to act on that sensation in a timely manner. In these scenarios, a wearable device that provides an alert as the bladder is approaching full could be very valuable in allowing users to void on time. Usage of such a tool would help prevent accidents and unnecessary catherization in patients with spinal cord injuries, help support toilet training in children with intellectual and developmental disabilities, and enable elderly in care homes to reach the toilet in time. Overall, bladder monitoring with proactive fullness alerts will reduce the incidence of UI-related side-effects, and would promote independence and overall well-being.

**Fig. 1.**
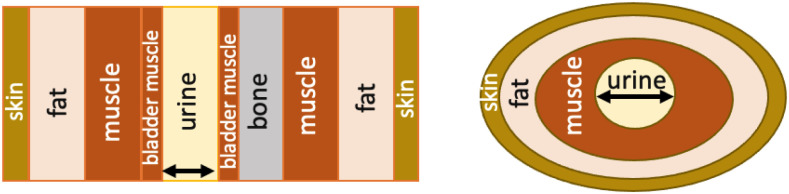
Most advanced layered models studied to date: (left) flat multi-layered structure (this one includes six tissue types, from [14]); and (right) elliptically-shaped multi-layered structure (including four tissue types, from [13]). The black arrows indicate variable urine volumes considered.

Microwave (MW)-based methods have strong potential to provide proactive detection of bladder state as a support tool for those with UI: they are safe for 24/7 use, non-invasive, low-cost, and could be made wearable [6]. Further, as the bladder fills with urine (which has higher dielectric properties than the bulk background tissues) it provides an increasing dielectric change in the pelvic region, leading to a growing target which is expected to modify the propagation of MW signals [6]. Some very preliminary studies on using MW-based approaches for bladder state detection have investigated the feasibility of this idea and shown promise; however, they have utilized simplified models having limited realism [7], [8], [9], [10], [11], [12], [13], [14], [15], [16] (see Fig. 1). Pelvic models, based on the Virtual Population models [17], were proposed in [18] for use in classification of the bladder state. These were the most advanced models proposed to date, based on human imaging data, and incorporated a wide range of tissues, including skin, blood vessels, bones, fat, intestines, and muscle. However, the full 3D models were not used in the study, which simulated only a 2D model with point source excitations, limiting the overall realism achieved. Notably, there has been no progress in this field in several years [6].

Overall, there is a notable gap in terms of studies investigating microwave bladder fullness with anatomically and dielectrically realistic pelvic models that can be readily used with computational electromagnetic (EM) solvers. The pelvic region is challenging due to its complexity and heterogeneous distribution of tissues, resulting in a difficulty in conducting simulations that are both time and memory-efficient. Further, research with these models have almost always included studies of changing the urine volume (i.e., changing the 2D or 3D dimensions of the bladder), but none to date have included the impact of uncertainty in urine properties. Additionally, no studies to date were conducted with antennas that could be practically used in a MW bladder monitor as they were focused on end-fire (e.g., Vivaldi-type) antenna designs [13], [14], [15].

This work addresses these gaps. We perform the most advanced realistic simulations to date to examine the technical feasibility of microwave bladder state detection. Specifically, we tackle three key gaps to advance the current state of this field. We first design and implement a realistic 3D model of both male and female pelvic regions to enable electromagnetic simulations that are as accurate as possible while still being achievable given today's limits of computational memory. We then simulate the models using a flexible antenna array (wherein each antenna has broadside radiation), which provides the first demonstration of a microwave bladder state detection system using an antenna type that is practically feasible. We simulate both male and female pelvic regions under scenarios of: varying bladder volume and varying urine conductivity. This work is the first time that a study has ever examined the impact of urine variability on bladder state detection. Moreover, given the use of the most realistic models to date combined with representative tissue properties, this study provides the most realistic examination of the feasibility of microwave bladder state monitoring to date - providing a proof-of-concept of bladder state detection through both examination of S-parameters and radar imaging results.

The article is structured as follows. In Section II, the design and implementation of anatomically and dielectrically representative computational models of the pelvic region are proposed; and the simulation methodology is described along with scenarios investigated and the procedures used for image reconstruction. The results and analysis are presented in Section III, along with a critical discussion of current challenges and potential future work in Section IV. Finally, the article concludes with Section V.

## II. Materials and Methods

In this section, the pelvic models are described in detail. Each of the chosen model parameters are indicated, along with the reasoning behind each design choice. The steps taken to optimize the models to enable simulations reasonable in both memory and time are detailed. Different considered scenarios are described. Lastly, the post-processing image reconstruction of bladder state is overviewed.

### A. Pelvic Model Preparation

The models for the pelvic region used in this work are based on the AustinMan and AustinWoman models [19], [20]. These models were developed based on the Visible Human Project [21], [22], [23] which consists of magnetic resonance imaging (MRI), computed tomography (CT), and other anatomical images. The AustinMan and AustinWoman models are voxel-based models with 1 × 1 × 1 $\text{mm}^{3}$ resolution. (We note that other highly-realistic voxel-based models have also been developed, e.g., from the IT'IS Foundation [24]; however the AustinMan and AustinWoman models are available to us with no restrictions on use, and we can modify them freely for use in any EM solver.)

We note that the male model is derived from a 38 year old with height of 71” and weight of 199 lb [21], resulting in a body mass index (BMI) of 27.8 kg/m^2^. The female model is from a 59 year old with BMI of 36 [25]. (Normal healthy adult BMI ranges from 18.5 to 24.9 kg/m^2^ [26]). Additionally, we previously estimated the tissue contents occupying the pelvic region for both male and female body models, and found that the male model is composed of approximately 34% fat, 42% muscle, and 24% other tissues, by volume occupied in the pelvic region, and the female model is approximately 49% fat, 26% muscle, and 25% other tissues [27]. Therefore, these models represent two very different test cases, with wide variations in tissue compositions.

**Fig. 2.**
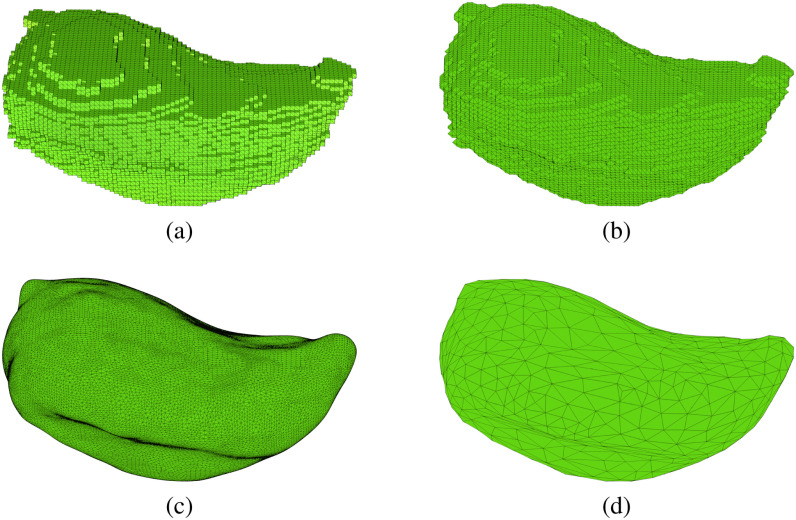
Process of converting voxel-based models to surface models. (a) Reference voxel model, (b) surface model obtained from marching cube algorithm, (c) smoothed model, and (d) final model with reduced elements used in the simulations.

Here, the electromagnetic simulations are based on the finite element method (FEM) which requires 3D volume models. In order to achieve a model that can be readily simulated, the full body of the AustinMan and AustinWoman models are first cropped so that only the torso region remains, including at least 5 cm above and below the height boundaries of the empty bladder. Then, the model was simplified by converting the tissues present into only a subset of key tissues. Next, employing the marching cube algorithm, the voxel-based models were converted to surface models. The surface models for each tissue were smoothed, and the number of surface elements reduced by coarsening the models where possible without losing the details of the anatomical structure. This process is shown in Fig. 2 for the male bladder.

### B. Selection of Model Parameters

In this section, we describe and justify the choice of each of our model parameters. The selection of tissues, assigned tissue properties, and dimensions are discussed.

#### 1) Tissues Included in the Model

The included tissues in this work are skin, fat, muscle, bone, bladder wall, and urine. As these tissues have relatively more volume than other tissues, as quantified in [27], they are expected to dominate microwave responses in the region.

#### 2) Dielectric Properties of Tissues

**Fig. 3.**
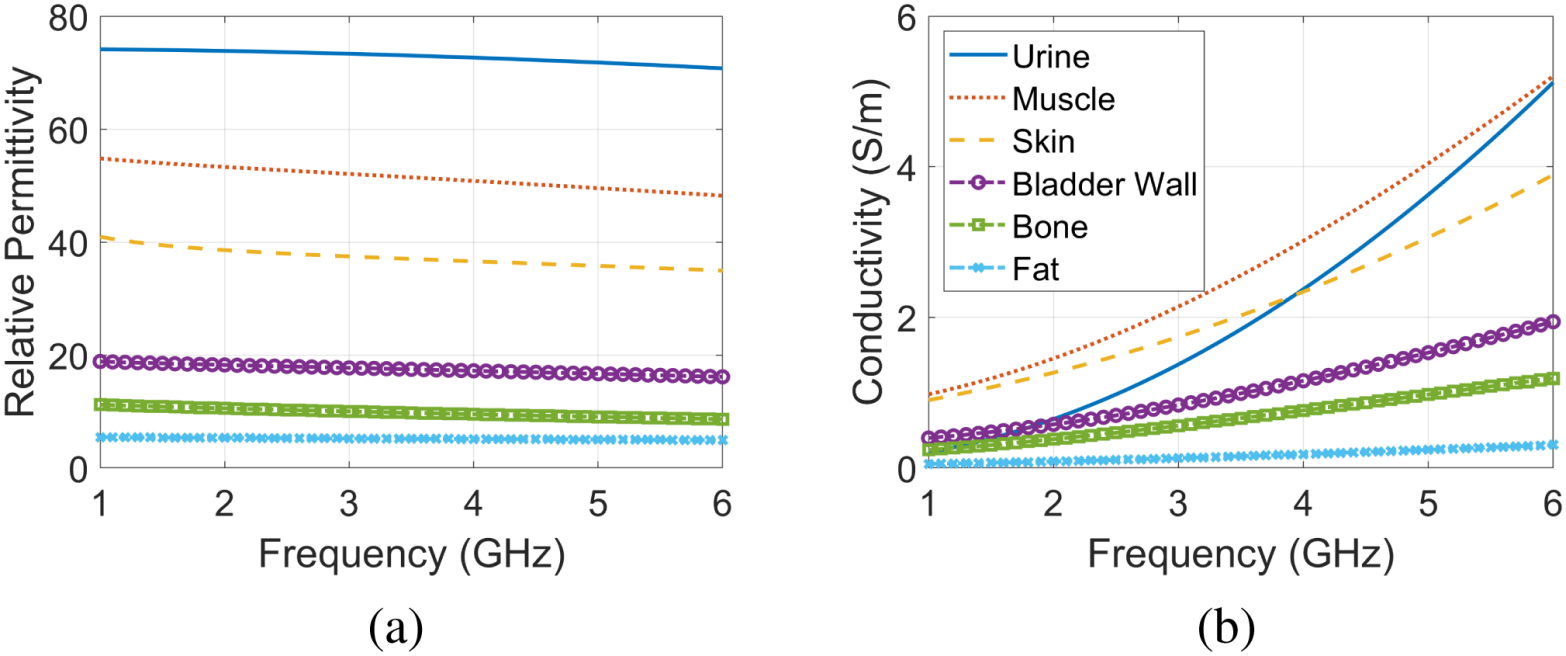
(a) Relative permittivity and (b) conductivity vs. frequency for the tissues included in the pelvic model: skin, fat, muscle, bone, and bladder wall data from [28], and urine data from [29].

The dielectric properties assigned to the skin, fat, muscle, bone, and bladder wall within the model are those from the IT'IS database [28]. Both the conductivity and relative permittivity are frequency-dependent. The relative permittivity and conductivity curves for each tissue included in the model are provided in Fig. 3.

#### 3) Dielectric Properties of Urine

**Fig. 4.**
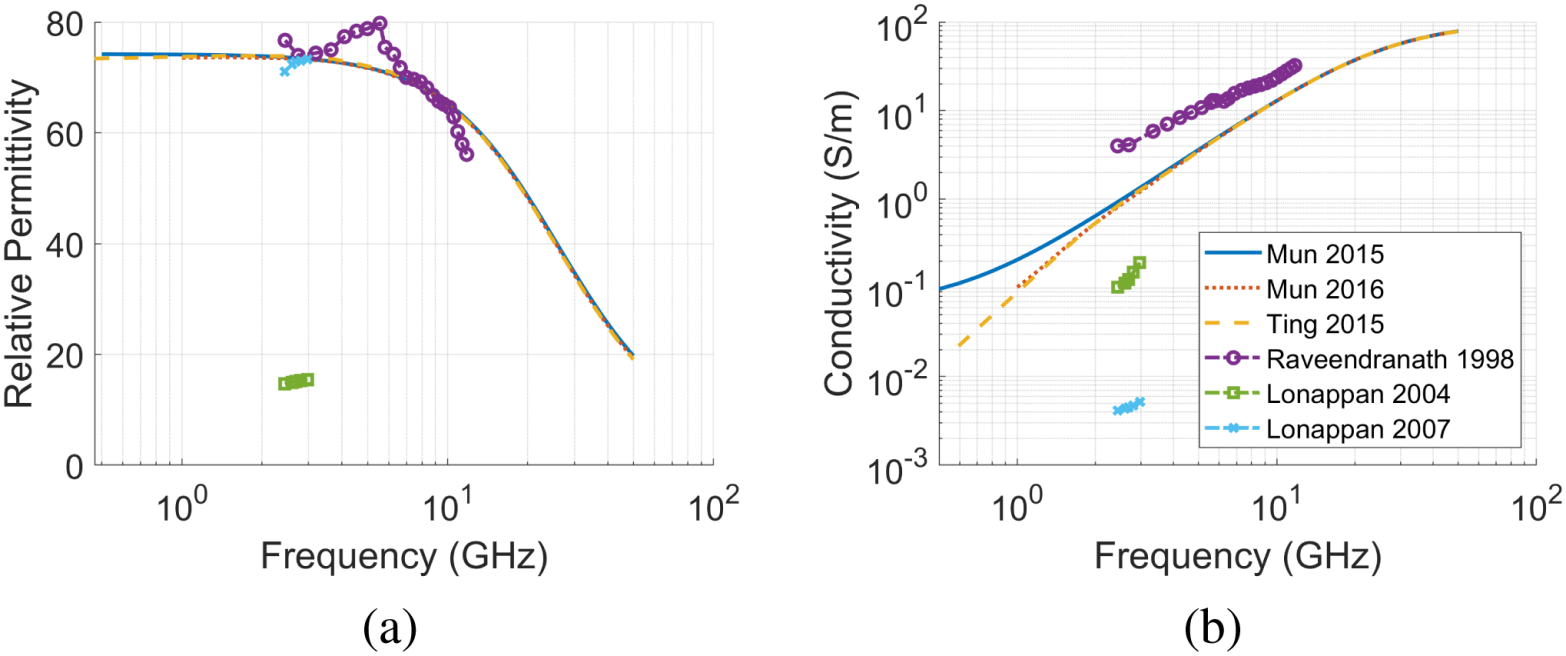
Measurements of the dielectric properties of human urine found in the literature. The mean values of (a) Relative permittivity and (b) conductivity are plotted at a temperature of 37 °C as reported in [29], [30], [31], [32], [33], [34].

The dielectric properties of urine are chosen based on literature reports of healthy adult urine samples. The relative permittivity and conductivity curves for urine are provided in Fig. 4. In [29], the dielectric properties of urine samples from 40 healthy subjects were recorded across our frequency range of interest. The mean values from [29] are consistent with those of [15], [30], [31], [32]. Two other studies, [33] (2 healthy volunteers) and [34] (4 healthy volunteers) also reported urine properties within our frequency range of interest. However, these have either significantly lower relative permittivity (about 80% lower) or conductivity values (>2 orders of magnitude lower) as compared to the first group of studies. As the dielectric properties of urine are expected to be dominated across this frequency range by the properties of water [35], higher relative permittivity values likely make sense (consistent with [29]). Thus, the mean dielectric properties of urine from [29] are chosen as the base properties to study in this work (as shown in Fig. 3 along with the tissue properties of the other tissues).

However, the conductivity of urine may vary significantly from person-to-person, and data on intra- or inter-individual variability is not available. Therefore, due to the inconsistency and unknowns in reported properties for human urine, we simulate four cases to add uncertainty to the urine dielectric properties in order to introduce inherent variability in these values. These cases include the lower and upper limits of the values reported in literature. Also, two cases with $\pm$20% variation from the upper limit case were considered to account for intra- or inter-individual variability. Specifically, we define case 1 using the mean dielectric properties from [29]. Case 2 and case 3 use the same definition with +20% and −20% uncertainty in both permittivity and conductivity, respectively. Lastly, case 4 uses the dielectric properties reported in [33].

#### 4) Bladder Volume and Filling

The empty bladder volume is approximately 50 ml for the male model and 30 ml for female model which is consistent with the reported values in the literature [36], [37]. However, these models do not provide full or half-full bladder cases. Additionally, no realistic models for dynamic bladder filling were found in the literature. Values as high as 900 ml are reported for full bladder [38]. However, the maximum when the body feels the need to void is reported around 500 ml [39], [40]. Therefore, the volume for the full and half-full bladder are assumed to be 470 ml and 235 ml for the male model and 335 ml and 130 ml for female model, respectively.

**Fig. 5.**
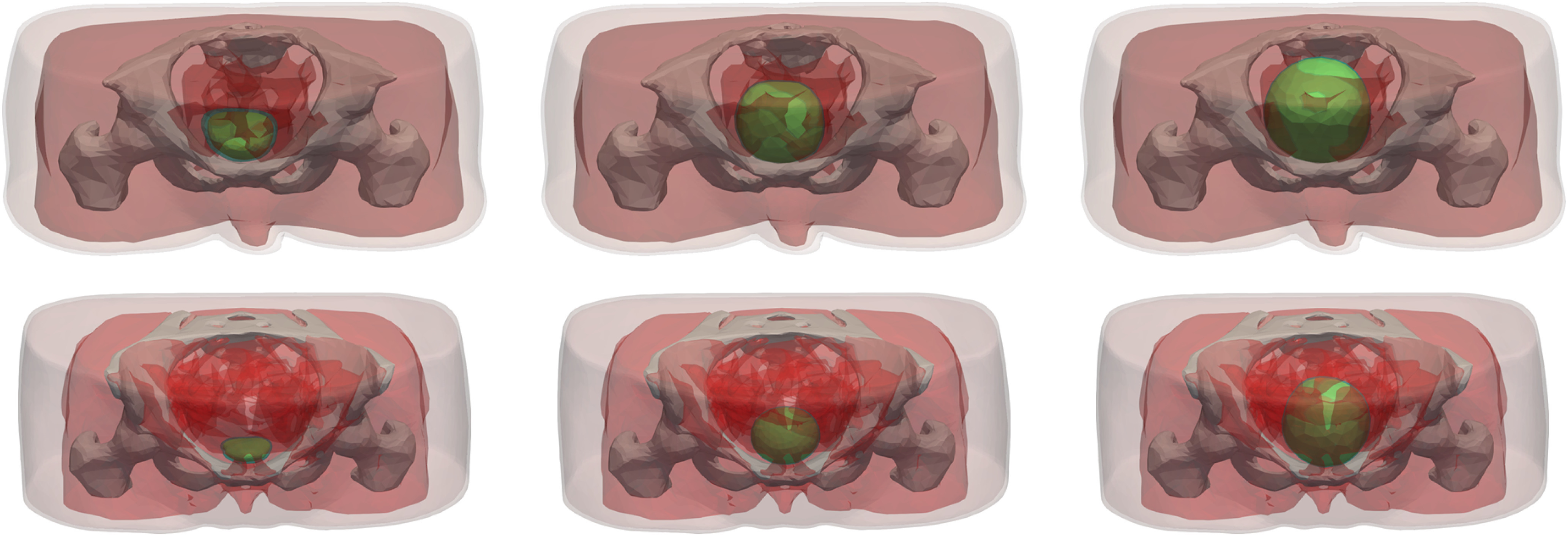
Full pelvic models, showing all constituent tissues. The top figures represent the male model and the bottom figures represent the female model. From left to right, empty, half-full and full bladder models are represented.

The models for bladders of different volumes were created by expanding the empty bladder model in all 3-dimensions at the same time, with different growth rates in each dimension, to achieve the required bladder volume. The expansion was based on the bladder growth models presented in [41], which were developed from MRI scans taken as the bladder filled. The bladder wall thickness in the models vary across the structure, with a maximum thickness of 2 mm, in both empty and full cases. The resulting pelvic models are shown in Fig. 5.

### C. Pelvic Model Optimization for Simulation

**Fig. 6.**
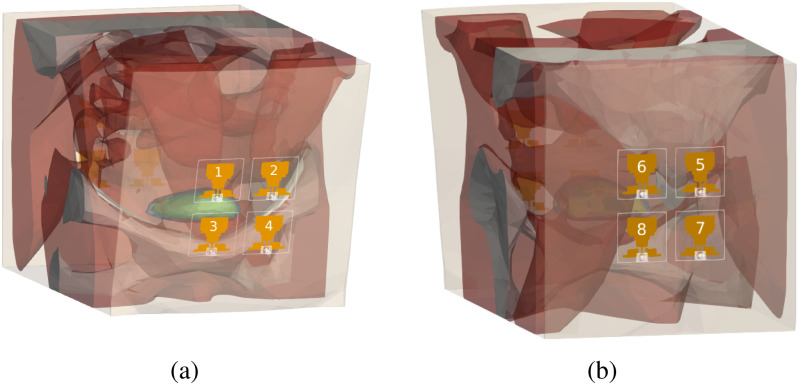
Antenna array on the (a) front of the female model, (b) back of the female model.

Initial simulations with a full, uncropped pelvic region model indicated that the complexity and detailed heterogeneity of the pelvic region was too memory intensive to simulate at high frequencies. To reduce the computational costs of the simulations, the size of the pelvic models were reduced by limiting the width of the models. An example of this simplification is shown in Fig. 6. The effects of this model simplification on accuracy of the simulations was studied in [42] using a single antenna placed in front of the pelvic region at 1 GHz. Based on [42], reducing the width of the model up to 100 mm has a negligible effect on reflection coefficient calculations. Since the width of the antenna substrate is 30 mm, considering a 35 mm wide region on each side of the antenna results in a negligible difference in reflection coefficient. This 35 mm margin is considered in all the simulations.

Time and memory costs of the simulations for both the full pelvic model and optimized model are presented in Table I for the male model. The simulations are done on a Intel Xeon Platinum 8160 node with 48 cores and 192 GB of RAM. In HFSS, the high performance computing (HPC) options were enabled using 48 cores for all the simulations. The full pelvic model simulations required more memory than is available for frequencies above 3 GHz. Note, similar memory limitations were faced with the female model.

**TABLE I:** Comparison of the Time and Memory Costs of the Simulations for Full Pelvic Model and Optimized Model

	Full Pelvic Model		Optimized Model	
Frequency	Time (sec)	Memory (GB)	Time (sec)	Memory (GB)
1 GHz	368	19.4	193	10.4
2 GHz	3952	111.0	456	27.5

### D. Simulation Set-Up

Simulations using the optimized pelvic models were done in HFSS using the 3D finite element method (FEM) at 81 frequency points between 1 and 5 GHz (50 MHz steps). Discretization was done automatically using adaptive mesh convergence in HFSS with a 0.01 threshold. The simulated model requires a radiation box to limit FEM solver's domain and this radiation box is added automatically by HFSS. The dielectric properties of the tissues were defined as frequency-dependent based on definitions given in Fig. 3. For excitation, the monopole antenna design from [43] was used and placed in contact with skin. The antenna was originally used for breast cancer detection and designed to match skin, fat and gland layers from 2 to 5 GHz. The dimensions of the antenna are given in detail in [43]. For the purpose of this work, the antenna was tested on a skin layer backed by fat and muscle layers and had a good match (reflection coefficient <−10 dB) between 1.5 to 5.5 GHz. Therefore, the antennas are placed in direct contact with the skin, with no need for a matching layer. The backside of the antennas are in air. Two 2 × 2 arrays of this antenna were used in the simulations, forming an eight-antenna configuration with one array on the front of each model and another one on the back of each model, as shown in Fig. 6. This results in 36 channels between the 8 antennas (i.e., 7 × 8/2 = 28 $S_{ij}$ channels between antennas and 8 $S_{ii}$ channels).

### E. Image Reconstruction

The Microwave Radar-based Imaging Toolbox (MERIT) [44] was used to reconstruct images of the pelvic region surrounding the bladder. MERIT is an open source MATLAB framework for developing and optimizing microwave imaging algorithms. MERIT reconstructs an image within a defined region, given the time or frequency domain back-scattered signals for different channels. It uses the Delay-and-Sum (DAS) algorithm to synthetically focus signals to the points within imaging domain. The focused signals from each channel are summed and the energy of the summed signal is used to reconstruct an energy profile of the imaging domain, highlighting areas of high dielectric contrast. The complex-valued S-parameters obtained from simulations are used as the input signals into the DAS algorithm.

## III. Results

First, the impact of bladder filling on detecting bladder volume is analyzed, followed by the impact of urine conductivity. Imaging results that demonstrate the feasibility of bladder state detection are also presented.

### A. Impact of Bladder Filling on S-Parameters

**Fig. 7.**
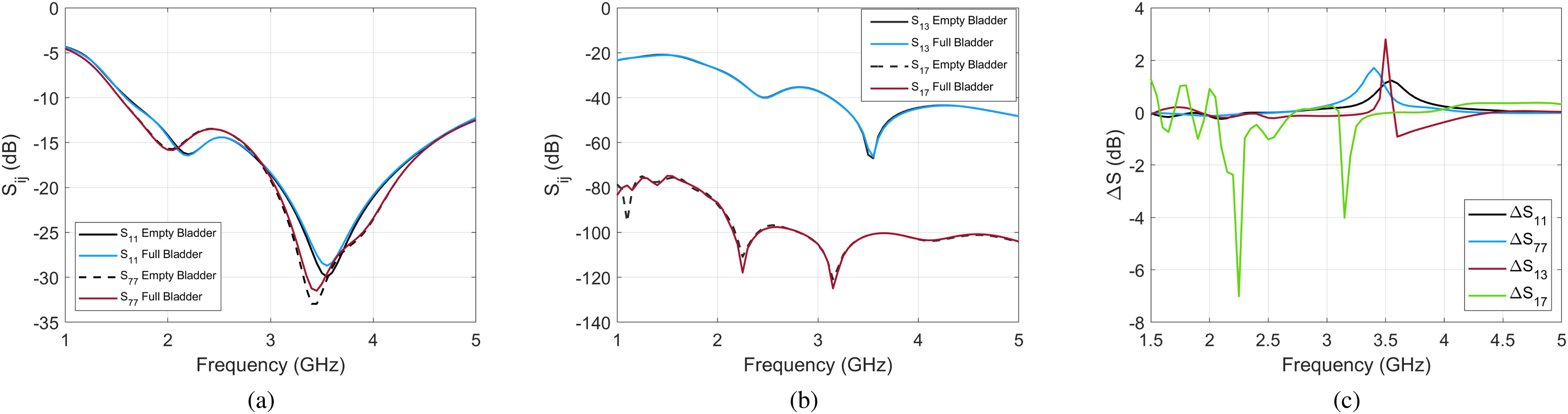
(a) Reflection coefficients ($S_{ii}$) for antennas 1 and 7, (b) transmission coefficients ($S_{ij}$) between antenna 1 and antennas 3 and 7, (c) the difference between the empty and the full bladder for these coefficients, for the male model. The order of antennas is shown in Fig. 6.

In this section, the nominal values for permittivity and conductivity given in Fig. 3 are considered for all the tissues. To quantify the effect of the bladder filling, the reflection coefficient of the antennas ($S_{ii}$) as well as the transmission between antennas ($S_{ij}$) are simulated for empty and full cases for both male and female models. With eight antennas, considering the reciprocity theorem, there are thirty six unique values for $S_{ij}$ for $i,j=1,{\ldots },8$ at each frequency. Fig. 7 plots example trends of these $S_{ij}$ values for a couple channels. To summarize all channels, the maximum and mean difference between the empty and full bladder states are also reported in Table II for different frequency ranges. While differences are not evident in every channel or at every frequency point, it is clear from the summary in Table II that as an overall dataset, differences are measurable.

**TABLE II:** Maximum and Mean Difference for Reflection and Transmission-Based $S_{ij}$ Between Full and Empty Bladder Cases

	$\Delta {S_{ij}}$ (dB)		$\Delta {S_{ij}}$ (dB)	
	Reflection-based		Transmission-based	
Frequency	Mean	Max	Mean	Max
1-2 GHz	0.064	0.472	2.738	33.008
	0.219	3.635	4.714	31.423
2-3 GHz	0.068	0.287	0.739	7.007
	0.381	3.241	2.315	25.862
3-4 GHz	0.265	2.798	0.278	4.017
	0.283	2.034	1.902	17.396
4-5 GHz	0.091	0.416	0.333	2.724
	0.418	1.969	1.116	6.951

‘Reflection-Based’ S-Parameters are Defined to Include Both $S_{ii}$ Data and $S_{ij}$ (I.e., $S_{ij}$ Where *i* = *J* is Possible), Where Antennas *I* and *J* are on the Same Side of the Body. Results of Male and Female Models are Represented as Blue and Red Cells, Respectively. We Note That the Standard Deviation of the Mean S-Parameter Difference, Over All Frequency Points, is 0.0013 for the Female Model and 0.0007 for the Male.

### B. Impact of Urine Permittivity on S-Parameters

**Fig. 8.**
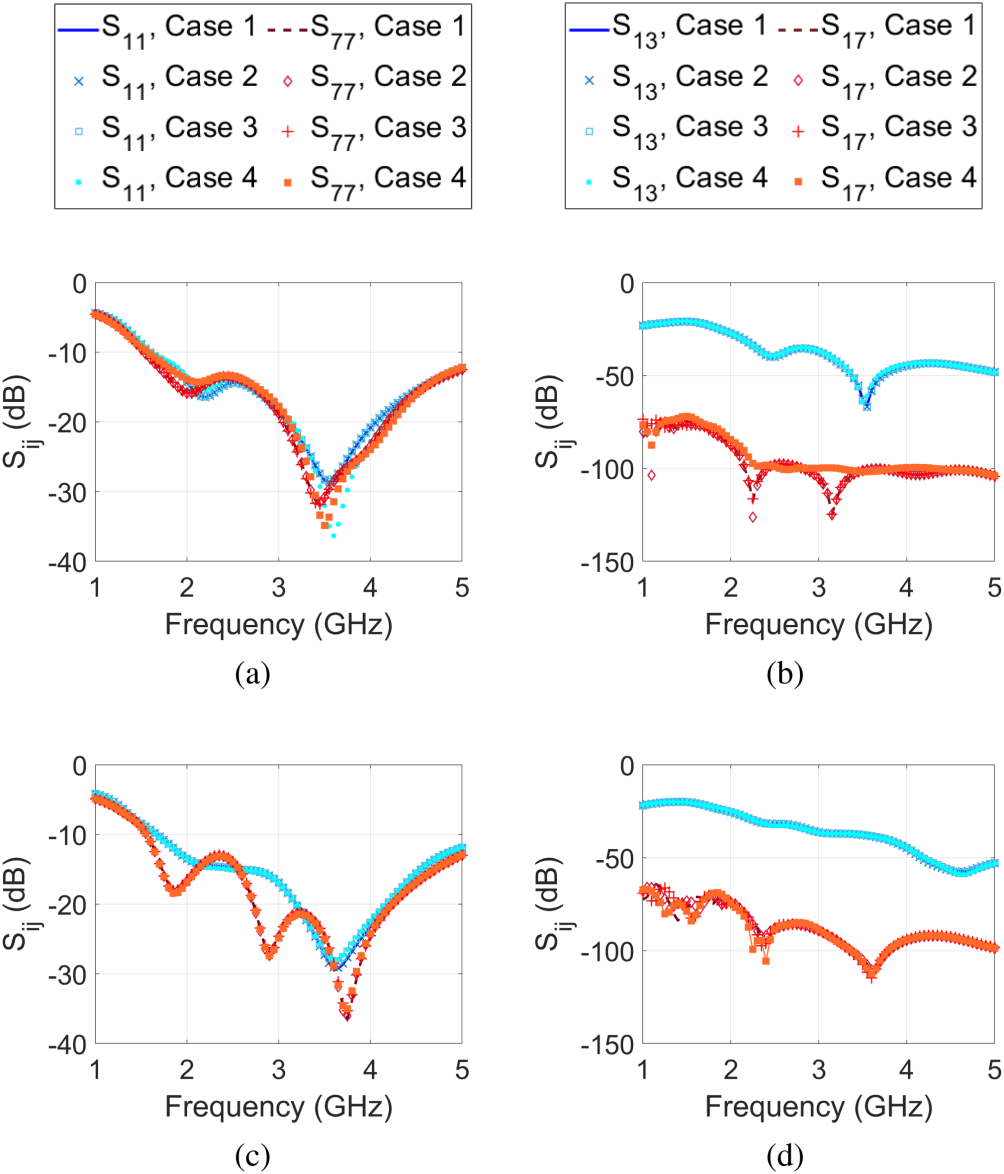
Comparison of S-parameters for four cases of different urine dielectric properties: case 1 uses the mean dielectric properties from [29]; cases 2 and 3 uses this mean $\pm$ 20% uncertainty, respectively; and case 4 uses the properties from [33]. Reflection coefficients for antennas 1 and 7 for (a) the male model and (c) the female model. Transmission coefficient between antenna 1 and antennas 3 and 7 for (b) the male model and (d) the female model.

Next, we compare the results for four cases of urine described in Section II-B3. First we consider the full bladder state and compare the results for these four cases. Fig. 8 shows the reflection and transmission coefficients for the selected antennas. Based on these results, $\pm$20% uncertainty (i.e., cases 2 and 3) does not have a significant effect on the reflection coefficient, and only has minor effects on the transmission coefficient at low frequencies. However, there are noticeable differences between case 1 and case 4 especially for the male model. Similar results were observed for the reflection and transmission coefficients of other antennas.

**Fig. 9.**
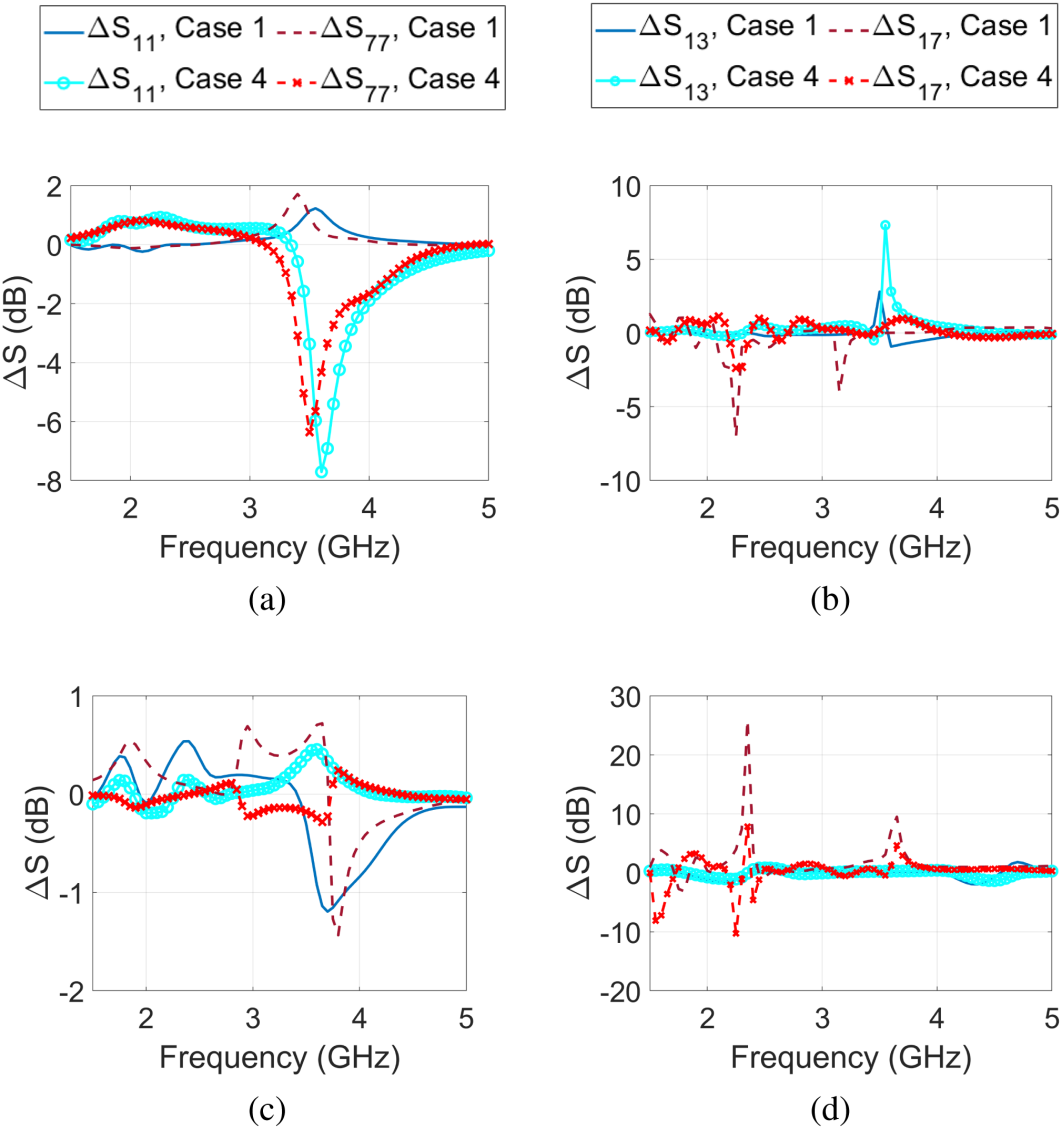
Difference of S-parameters between empty and full bladder for different urine dielectric properties: case 1 (from [29]) and case 4 (from [33]). Reflection coefficients difference for antennas 1 and 7 for (a) the male model and (c) the female model. Transmission coefficient difference between antenna 1 and antennas 3 and 7 for (b) the male model and (d) the female model.

Finally, to see the effect of dielectric properties of urine in detectability of bladder state, the results for case 1 and case 4 are compared for full and empty bladder cases. Fig. 9 shows the difference between the empty and full bladder for case 1 and case 4. This data shows that urine permittivity may impact detectability of bladder fullness.

### C. Image Reconstruction

**Fig. 10.**
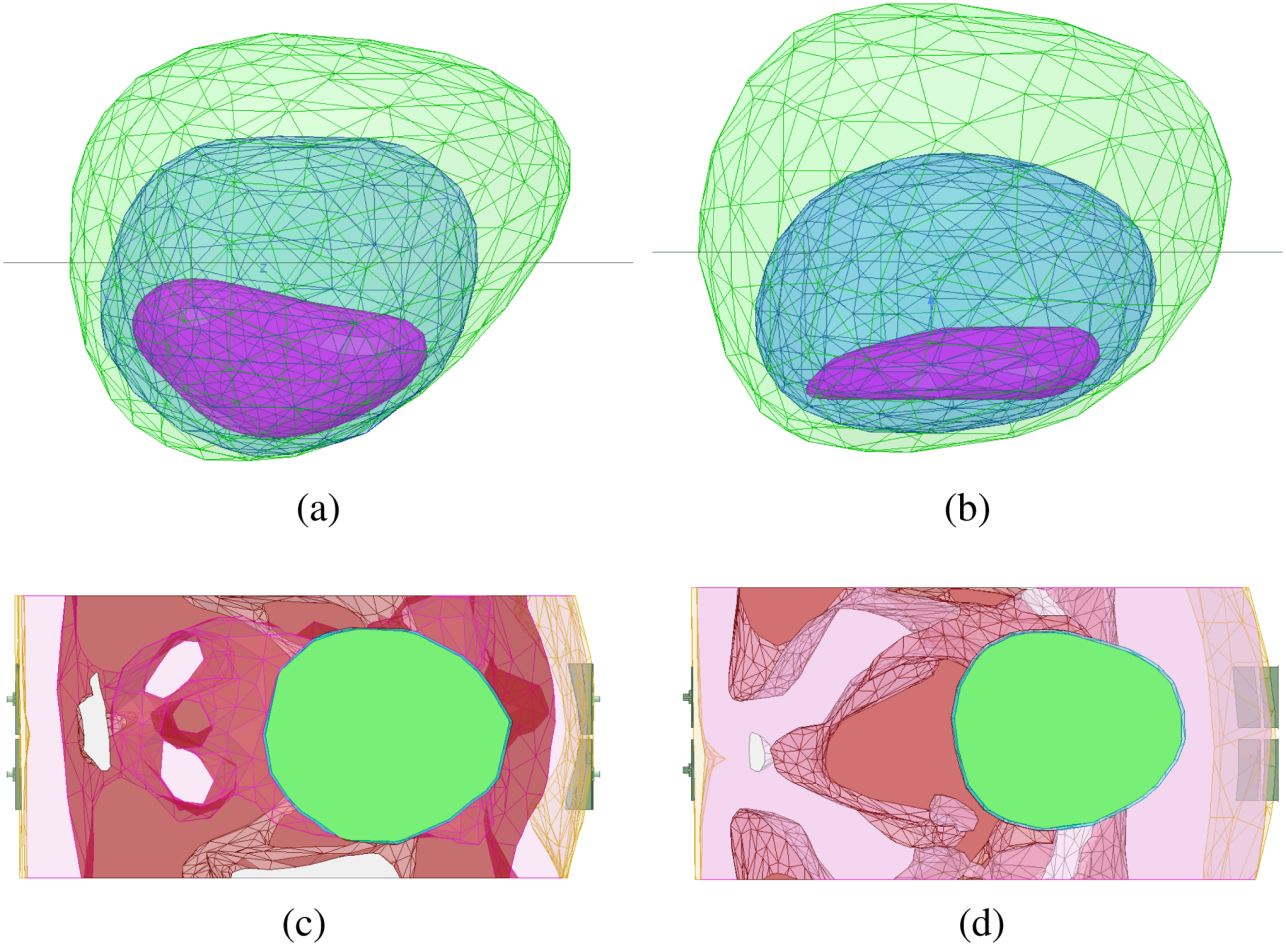
Side view of empty, half-full, and full bladder for (a) male and (b) female models. The image reconstruction domain of the (c) male, and (d) female models (top view). The relative location of the imaging plane with respect to bladder is shown as a black line in (a) and (b).

**Fig. 11.**
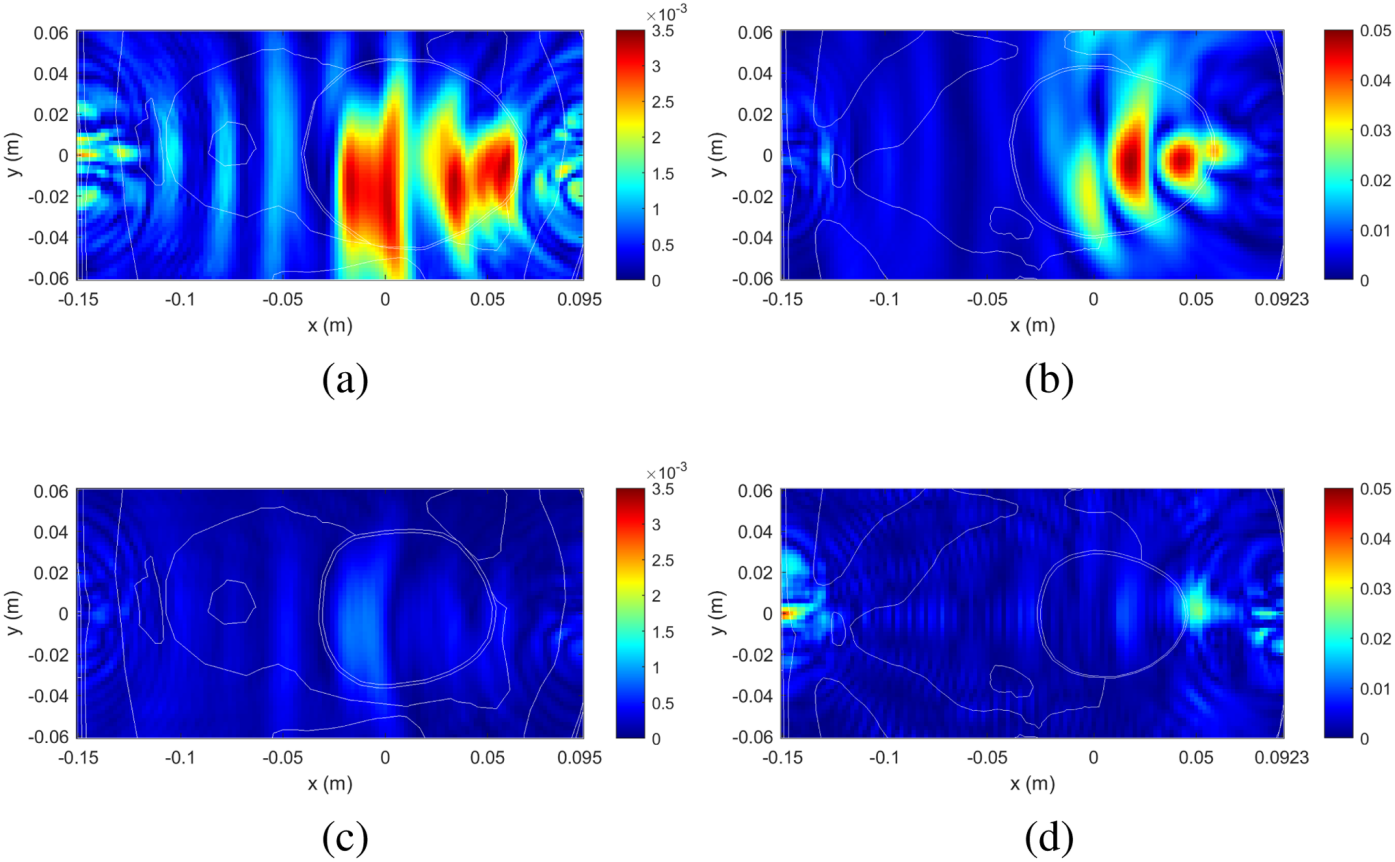
Reconstructed image of a slice of the pelvic model at $z = 20$ mm. Full bladder compared to empty bladder for (a) male and (b) female models. Half-full bladder compared to empty bladder for (c) male and (d) female models. The images include an overlay of the cross-section of the pelvic model, as seen in Fig. 10 (which has the same orientation and approximate dimensions), in order to indicate the location of the bladder.

The imaging domain is selected as a rectangular grid at $z = 20$ mm with a resolution of 2 mm including all of the simulation domain in $xy$ plane. The relative location of the imaging domain with respect to the bladder is shown in Fig. 10. The full and half-full bladder cases are compared to the empty bladder case, with reconstructed images shown in Fig. 11. In these radar images, red indicates regions of high scattering and blue indicates areas of weak scattering. We see that the scattering from the bladder is highest with a full bladder and is minimal with a bladder volume that is more empty. In both male and female models, clear differentiation of full and empty bladders is apparent.

## IV. Discussion

In general, the results indicate the potential for bladder fullness detection using microwave measurements. The results further suggest that transmission-based measurements may contain more valuable information for discriminating bladder volume than reflection-based ones. However, we note that reflection-based measurements, which result in higher $S_{ij}$ values, are likely to be more feasible than transmission-based measurements for a practical wearable device: reflection-based measurements result in higher $S_{ij}$ values which would be more robust in detection even with noise sources present, and, front-of-body arrays would likely be easier for an individual to place and consistently position than back-of-body ones.

Additionally, considering the varying results for the male and female models, the efficiency of MW-based methods are likely affected significantly by the body mass index (BMI)/tissue content in the pelvic region. It is important to keep in mind that these models represent only one male and one female body, and it is expected that there will be large inter-individual variation in body size and composition, which should be accounted for in future studies. These methods might work better for child-based applications, e.g., support of nocturnal enuresis, where there is less loss because of smaller volume of the tissues.

However, overall, the results have demonstrated potential for bladder state monitoring or full bladder detection. We note that this has been only a preliminary study of the potential of this application and further work will be needed to optimize a MW prototype for this use. Considering different array configurations may result in more robust detectability of bladder state. The antenna type may have a significant effect on the results as well, along with how the antenna is placed on the body (i.e., with an adhesive layer like a band-aid or embedded in clothing). The optimal frequency point or range also remain to be identified. Furthermore, future work could include the investigation of binary classifiers to classify full vs. not-full bladder state. Even simple thresholding approaches may be usable in some scenarios. The impact of varying urine conductivities, body sizes and shapes should also be studied.

## V. Conclusion

In this work, microwave bladder state detection was studied using the most realistic pelvic models to date. A realistic antenna array design was used to excite the models, which were simulated in 3-dimensions. The results, examined through changes in S-parameters and reconstructed images indicate the potential of using MW-based approaches for bladder state detection.

While the results show potential for this approach, future work to optimize numerous parameters (e.g., antenna type and positions, frequency range), may further improve MW bladder state detection. Lastly, pilot studies with humans are required to definitively demonstrate the usability of MW bladder monitoring in the real world.
